# Draft Genome Sequences of Amoeba-Resistant *Aeromonas* spp. Isolated from Aquatic Environments

**DOI:** 10.1128/genomeA.01115-14

**Published:** 2014-10-30

**Authors:** Takehiko Kenzaka, Mayu Nakahara, Shoko Higuchi, Kento Maeda, Katsuji Tani

**Affiliations:** Environmental Science and Microbiology, Faculty of Pharmacy, Osaka Ohtani University, Nishikiori-kita, Tondabayashi, Japan

## Abstract

Amoeba-resistant *Aeromonas veronii* ARB3 and *Aeromonas media* ARB13 and ARB20, which may be important intracellular pathogens of eukaryotic hosts, were isolated from pond and river waters. The draft genome sequences indicate that the strains harbor multiple protein secretion systems and toxins that induce disruption of the actin cytoskeleton.

## GENOME ANNOUNCEMENT

The genus *Aeromonas* has been isolated from diverse aquatic environments, including rivers, lakes, ponds, seawater (estuaries), drinking water, and groundwater (1). Some species are virulent in various living organisms, such as protozoa, worms, fish, mice, and humans (2–4). Thus, assessing the virulence of *Aeromonas* bacteria is challenging with respect to their clinical importance (5).

The interactions between free-living amoebae and parasitic bacteria exhibit similar processes to those that occur during the infection of mammalian cells by intracellular pathogens (6). Thus, amoebae may represent a good model for the development of resistance to macrophage digestion, which is often required for mammalian infection, and they represent environmental reservoirs of intracellular pathogens. The genetic organization of amoeba-resistant bacteria may provide new insights into the host–microbe interactions of potential human pathogens (7). We isolated amoeba-resistant strains *A. veronii* ARB3 from pond water and *A. media* ARB13 and ARB20 from river water, where *Acanthamoeba castellanii* ATCC 30234 was the host, and we present their draft genomes.

The draft genomes were sequenced by 100-bp paired-end sequencing on an Illumina Hiseq2000 sequencing system (Hokkaido System Science Co. Ltd., Sapporo, Hokkaido, Japan). High-quality sequence reads (23,819,920; 23,697,747; and 23,523,332) were assembled *de novo* using CLC Genomics Workbench v6.5 (CLCbio, Cambridge, MA, USA). The sequenced reads were mapped again to the contigs. Approximately 98.8% to 99.1% of the base reads were mapped to the updated contigs. The final assembly of the genome produced 4,542,657 bp in 63 contigs with an average length of 72,106 bp and a 58.8% G+C content for ARB3; 4,612,357 bp in 180 contigs with an average length of 25,624 bp and a 61.0% G+C content for ARB13; and 4,620,096 bp in 185 contigs with an average length of 24,973 bp and a 61.0% G+C content for ARB20. The assembled contigs were functionally annotated using the RAST annotation server (8). The genomes contained 4,017 putative coding sequences (CDSs) (76.9%) and 87 tRNA and rRNA genes for ARB3, 4,200 putative CDSs and 96 tRNA and rRNA genes for ARB13, and 4,216 putative CDSs and 99 tRNA and rRNA genes for ARB20.

The genomes of the amoeba-resistant *Aeromonas* spp. encode several potential colonization and virulence factors, particularly secretion systems and effectors. All three strains possess the type I secretion system (T1SS) for aggregation; T1SS-secreted agglutinin repeat-in-toxin (RTX) toxins, which cause cell rounding and actin depolymerization by covalently cross-linking actin monomers into multimers (9); the type II general secretion system (T2SS) and hemolysin; and the effector VgrG, which disrupts the actin cytoskeleton via ADP ribosylation of actin (10). T2SS is responsible for secreting hemolysin into the extracellular space in *A. hydrophila* (11). ARB3 also possesses the type III secretion system (T3SS) and type VI secretion system (T6SS). Both T3SS and T6SS are known to be important for virulence in *Aeromonas* spp. (3, 10, 12). The secreted toxins may disrupt the actin cytoskeleton in amoeboid cells.

### Nucleotide sequence accession numbers.

The draft genome sequences of the *Aeromonas* strains have been deposited in the DDBJ/EMBL/GenBank under the accession numbers JRBE00000000, JRBF00000000, and JRBG00000000.
